# Adaptation of a Chytrid Parasite to Its Cyanobacterial Host Is Hampered by Host Intraspecific Diversity

**DOI:** 10.3389/fmicb.2018.00921

**Published:** 2018-05-08

**Authors:** Ramsy Agha, Alina Gross, Thomas Rohrlack, Justyna Wolinska

**Affiliations:** ^1^Department of Ecosystem Research, Leibniz-Institute of Freshwater Ecology and Inland Fisheries, Berlin, Germany; ^2^Institute of Biology, Freie Universität Berlin, Berlin, Germany; ^3^Faculty of Environmental Sciences and Natural Resource Management, Norwegian University of Life Sciences, Ås, Norway

**Keywords:** algae, attenuation, genetic diversity, phytoplankton, *Planktothrix*, *Rhizophydium*, serial passage, transmission

## Abstract

Experimental evolution can be used to test for and characterize parasite and pathogen adaptation. We undertook a serial-passage experiment in which a single parasite population of the obligate fungal (chytrid) parasite *Rhizophydium megarrhizum* was maintained over a period of 200 days under different mono- and multiclonal compositions of its phytoplankton host, the bloom-forming cyanobacterium *Planktothrix*. Despite initially inferior performance, parasite populations under sustained exposure to novel monoclonal hosts experienced rapid fitness increases evidenced by increased transmission rates. This demonstrates rapid adaptation of chytrids to novel hosts and highlights their high evolutionary potential. In contrast, increased fitness was not detected in parasites exposed to multiclonal host mixtures, indicating that cyanobacterial intraspecific diversity hampers parasites adaptation. Significant increases in intensity of infection were observed in monoclonal and multiclonal treatments, suggesting high evolvability of traits involved in parasite attachment onto hosts (i.e., encystment). A comparison of the performance of evolved and unevolved (control) parasite populations against their common ancestral host did not reveal parasite attenuation. Our results exemplify the ability of chytrid parasites to adapt rapidly to new hosts, while providing experimental evidence that genetic diversity in host populations grants increased resistance to disease by hindering parasite adaptation.

## Introduction

In recent years, it has become evident that the impact of disease goes beyond direct effects on host abundance. Parasites (used here to refer generically to pathogens, parasites, and parasitoids) can modulate large-scale nutrient and carbon cycles (e.g., Suttle, 2007), establish alternative trophic links (e.g., Agha et al., 2016), and promote genetic diversity (e.g., Turko et al., 2018). The antagonistic interaction between host and parasite is among the most intense selective pressures in nature, which manifests as an evolutionary arms race of reciprocal adaptations (Thompson, 1998; Woolhouse et al., 2002). In this race, parasites are usually ahead and are expected to adapt rapidly to their hosts, as they often show higher evolutionary rates (Gandon and Michalakis, 2002). Parasite adaptation to novel hosts frequently leads to reduced infectivity against former ones, causing attenuation (Ebert, 1998). Understanding the evolutionary trajectories of parasite adaptation (and attenuation) is critical to predicting patterns of local adaptation (Kawecki and Ebert, 2004; Greischar and Koskella, 2007), the success of biological invasions (Lafferty and Kuris, 1996; Torchin and Mitchell, 2004; Dunn, 2009), and the emergence of zoonotic diseases (Woolhouse et al., 2005). Since faster evolving parasites are expected to rapidly adapt and counteract host defensive innovations, hosts populations might be selected toward diversification in order to resist disease, as every host genotype constitutes a unique environment for the parasite (Altermatt and Ebert, 2008; Whitehorn et al., 2011). Due to the often devastating effects of disease outbreaks in monospecific crops (Leonard, 1969; Garrett and Mundt, 1999), the idea that host genetic diversity protects from disease has received a great deal of attention in agricultural research (e.g., Shipton, 1977; Sumner et al., 1981), but has rarely been tested directly in microbial communities.

Co-evolution between host and parasite can be studied by means of experimental evolution (Kawecki et al., 2012; Brockhurst and Koskella, 2013). This approach allows evolutionary effects on host and parasite to be disentangled, by conducting asymmetric serial passage assays, where one of the antagonists is allowed to evolve, while the other remains in evolutionary stasis. By restricting the evolution of the host, parasites can be maintained over a number of generations under novel host environments and the performance of evolved and ancestral parasite populations can be tracked in real time (Jinks and Grindle, 1963; Rubin et al., 1993; Meaden and Koskella, 2017). Thereby, such experiments provide unequivocal evidence of parasite and/or host adaptation. Organisms with short generations, such as phytoplankton, are most amenable to study evolution through serial passage experiments. Phytoplankton constitutes the base of most aquatic food webs and drives major biogeochemical cycles, e.g., phytoplankton is responsible for 50% of global carbon fixation (Falkowski, 2012). Phytoplankton is known to be attacked by different parasites (e.g., viruses, bacteria, fungi; Park et al., 2004; Gachon et al., 2010; Gerphagnon et al., 2015), but the evolutionary feedbacks between phytoplankton and their parasites have been studied almost exclusively with regard to viral infections. For instance, viruses have been shown to induce rapid diversification of the widespread marine cyanobacterium *Synechococcus*, as a result of co-evolution (Marston et al., 2012).

In addition to well-studied viruses, recent molecular surveys have revealed a so-far disregarded diversity of ubiquitous fungal parasites that infect all major phytoplankton groups, most of which belong to the early diverging phylum Chytridiomycota (hereafter referred to as chytrids; see e.g. Lefèvre et al., 2008; Comeau et al., 2016; Gutiérrez et al., 2016). Among the profound effects of chytrid parasitism on phytoplankton populations and aquatic ecosystems as a whole (reviewed in Frenken et al., 2017), chytrids' evolutionary interactions with their phytoplankton hosts remain poorly studied. A single previous study experimentally demonstrated the ability of a chytrid parasite infecting the diatom *Asterionella formosa* to adapt to new genetically homogeneous hosts, but not to genetically heterogeneous host mixtures (De Bruin et al., 2008). This was interpreted as an indication that genetic diversity in host populations protects against disease (De Bruin et al., 2008). This notion is supported by field data suggesting that chytrid parasitism might promote genetic diversity in diatom host populations (Gsell et al., 2013). Still, further evidence seems necessary to link the high intraspecific genetic diversity typically found in natural phytoplankton populations with protection against disease. In particular, populations of prokaryotic phytoplankton (i.e., cyanobacteria) are characterized by remarkable genomic (e.g., Meyer et al., 2017) and metabolomic polymorphisms, including the production of toxins raising public health concerns and other bioactive peptides (Kurmayer et al., 2016; Haruštiaková and Welker, 2017). It is commonly assumed that intraspecific polymorphic traits confer on cyanobacteria increased versatility within and across ecological niches (e.g., Johnson et al., 2006; Rohrlack et al., 2008; Agha et al., 2014). Still, although hypothesized (Sønstebø and Rohrlack, 2011; Agha and Quesada, 2014), a link between cyanobacterial intraspecific diversity and increased resistance to fungal parasites has not been established directly.

In this work we aimed to evaluate (1) the ability of chytrids to adapt to host genotypes not previously exposed to the parasite (onwards referred to as new suboptimal host environments) and (2) whether host genetic diversity hampers parasite adaptation. To do so, we undertook a serial passage experiment, where a single parasite population was maintained over a period of 200 days under different mono- and multiclonal host compositions. We assessed the performance of each evolving parasite population over time, both against their respective new host environments (testing for parasite adaptation) and against their common ancestral host, i.e., host genotype on which the parasite is routinely maintained in culture (testing for parasite attenuation). We measured different parasite traits to evaluate which ones are more prone to evolve in response to novel host challenges. First, parasite transmission rate was calculated as a proxy for overall parasite fitness. Secondly, intensity of infection was scored to assess the ability of the parasite to find and successfully adhere to the host and, thereby, evade its barrier defenses. Lastly, the size of mature fungal reproductive structures (i.e., sporangia) was recorded as a proxy of parasite per capita reproductive output (i.e., greater sporangial sizes arguably imply more zoospores being released upon maturation) that negatively correlates with the intensity of infection (Agha et al., 2018).

## Materials and methods

### Host and parasite strains

The chytrid parasite strain Chy-Kol2008 and seven cyanobacterial host strains were used (Table S1). The parasite was previously identified as *Rhizophydium megarrhizum* (Sønstebø and Rohrlack, 2011). As with other chytrids, *R. megarrhizum* is characterized by presenting free-swimming infective stages in the form of flagellated zoospores that actively seek suitable hosts in the water column. Upon encystment, chytrids penetrate the host and extract nutrients from it, always leading to host death. Over the course of the infection, encysted zoospores develop into sporangia, reproductive structures that release asexually-produced zoospores upon maturation.

Host strains belong to the filamentous, bloom-forming, toxin-producing cyanobacterial genus *Planktothrix* (Table S1). *P. rubescens* (one strain) and *P. agardhii* (six strains) are, according to current taxonomy, affiliated to different species based on their pigmentation (*P. rubescens* contains the photosynthetic red pigment phycoerithrine, while *P. agardhii* lacks it and shows green pigmentation). However, based on their high genetic similarity (Humbert and Le Berre, 2001) and the fact that red pigmentation has been shown to be acquired by horizontal gene transfer (Tooming-Klunderud et al., 2008), all strains are considered here conspecific. All cyanobacterial strains were maintained in Z8 medium (Kotai, 1972) as non-axenic batch cultures under 16°C and constant light of 15 μmol photons m^−2^ s^−1^. Previous analyses showed that all host strains could be successfully infected by the parasite strain (data not shown). Individual cyanobacterial strains used represent distinct genotypes, as evidenced by different patterns of oligopeptide production (Table 1; see next section for details on oligopeptide analysis). A sympatric and an allopatric strain were selected as monoclonal treatments to explore if adaptation potential might be influenced by the origin of the host: Strain NIVA-CYA630 and the chytrid parasite were isolated from nearby lakes (between which gene flow exists, Kyle et al., 2015). Conversely, NIVA-CYA588 was isolated from a German lake and arguably had not any previous contact with the parasite. The selection of NIVA-CYA588 among the other German strains used was entirely random.

**Table 1:** Intracellular oligopeptide compositions of the cyanobacterial strains used.

**Oligopeptide**	**Molecular mass [M+H]^+^**	NIVA-CYA98^*^	NIVA-CYA630^*^	**NIVA-CYA557**	**NIVA-CYA562**	**NIVA-CYA578**	**NIVA_CYA580**	**NIVA-CYA588**
Aeruginosin	593.5	+		n.a	n.a	n.a	n.a	n.a
Aeruginosin A	617.5	+		n.a	n.a	n.a	n.a	n.a
Oscillaginin B	581.5	+		n.a	n.a	n.a	n.a	n.a
Oscillaginin A	615.5	+		n.a	n.a	n.a	n.a	n.a
Anabaenopeptin C	809.6		+	n.a	n.a	n.a	n.a	n.a
Me-Anabaenopeptin C	823.6		+	n.a	n.a	n.a	n.a	n.a
Anabaenopeptin B	837.6	+	+	+		+	+	+
Anabaenopeptin A	844.6	+		+		+		+
Anabaenopeptin F	851.6	+	+			+	+	+
Oscillamid Y	858.6	+				+		+
Microcystin desmethyl LR	981.6	+	+	+		+		+
Microcystin desmethyl RR	1024.7	+	+	+		+		+
Microcystin desmethyl YR	1031.7		+					
Microcystin YR	1045.6							+
Oscillapeptin G	1112.7	+		n.a	n.a	n.a	n.a	n.a
Cyanopeptolin	1126.7		+	n.a	n.a	n.a	n.a	n.a
Oscillatorin	1240.4	+		n.a	n.a	n.a	n.a	n.a
Putative microviridin	1854.8		+	n.a	n.a	n.a	n.a	n.a
Putative microviridin	1971.8	+		n.a	n.a	n.a	n.a	n.a

Strains marked with an asterisk (i.e., Norwegian strains) were analyzed elsewhere for all oligopeptides (Rohrlack et al., 2009) and are reported in the table. The other strains (i.e., German strains) were analyzed for anabaenopeptins and microcystins only (see methods); n.a.: not analyzed

The parasite was maintained by transferring zoospore suspensions into uninfected cultures of the cyanobacterium *P. rubescens* NIVA-CYA98 (Table S1) every 2 weeks. Considering that this cyanobacterial strain has been used to maintain the parasite since isolation (i.e., 9 years), it will henceforth be referred to as “original host.” Previous experimentation with this host-parasite system indicates that the generation time of the chytrid *Rhizophydium megarrhizum* (defined as the period between the addition of zoospores to a healthy host culture and the observation of empty sporangia in the culture) is approximately 1–1.5 days at 20°C (unpubl. data).

### Analysis of oligopeptides compositions in cyanobacterial strains

Intracellular oligopeptide compositions were used as phenotypic markers to characterize the diversity of the cyanobacterial strains used. Since the production of individual oligopeptides is constitutively regulated and thus solely determined by the presence or absence of their respectively-encoding gene clusters (Welker and von Dohren, 2006), different patterns in oligopeptide production imply genetic differences among strains. Oligopeptide compositions of the cyanobacterial strains isolated from Norwegian lakes (i.e., strains NIVA-CYA98 and NIVA-CYA630) were analyzed as reported elsewhere (Rohrlack et al., 2009; Sønstebø and Rohrlack, 2011). For the remaining cyanobacterial strains, anabaenopeptins and microcystins were extracted from filters with freeze dried *Planktothrix* using 50% methanol. Detection and identification of oligopeptides was done using liquid chromatography mass spectroscopy (LC-MS/MS). The instrumental setup included a Waters Acquity Ultra-Performance Liquid Chromatography (UPLC) System equipped with a Waters Atlantis C18 column (2.1 × 150 mm, 5 μm particle size) and directly coupled to a Waters Quattro Premier XE tandem quadrupole MS/MS detector (Waters Norge, Oslo, Norway). The UPLC system was set to deliver a linear gradient from 10 to 45% acetonitrile in water (both acidified with 0.1% formic acid) within 10 min at a flow rate of 0.25 mL min^−1^. The column and auto sampler temperatures were 20 and 4°C, respectively. The MS/MS detector was run in positive electrospray mode (ESI+). Other general settings included a source temperature of 120°C, a desolvation temperature of 350°C, a drying gas flow rate of 800 L h^−1^, a gas flow at the cone of 50 L h^−1^, and standard voltages and energies suggested by the manufacturer for the ESI+ mode. Only the cone voltage and the settings for the collision cell were adapted to the respective compounds using in-house reference material. Nitrogen, continuously delivered by a nitrogen generator (model NG, Parker Balston, Haverhill, MA, USA), served as drying, nebulizing, and cone gas. Anabaenopeptins and microcystins were identified by comparing fragmentation patterns of suspected oligopeptides with those of in-house reference material.

### Experimental setup

**Figure 1:**
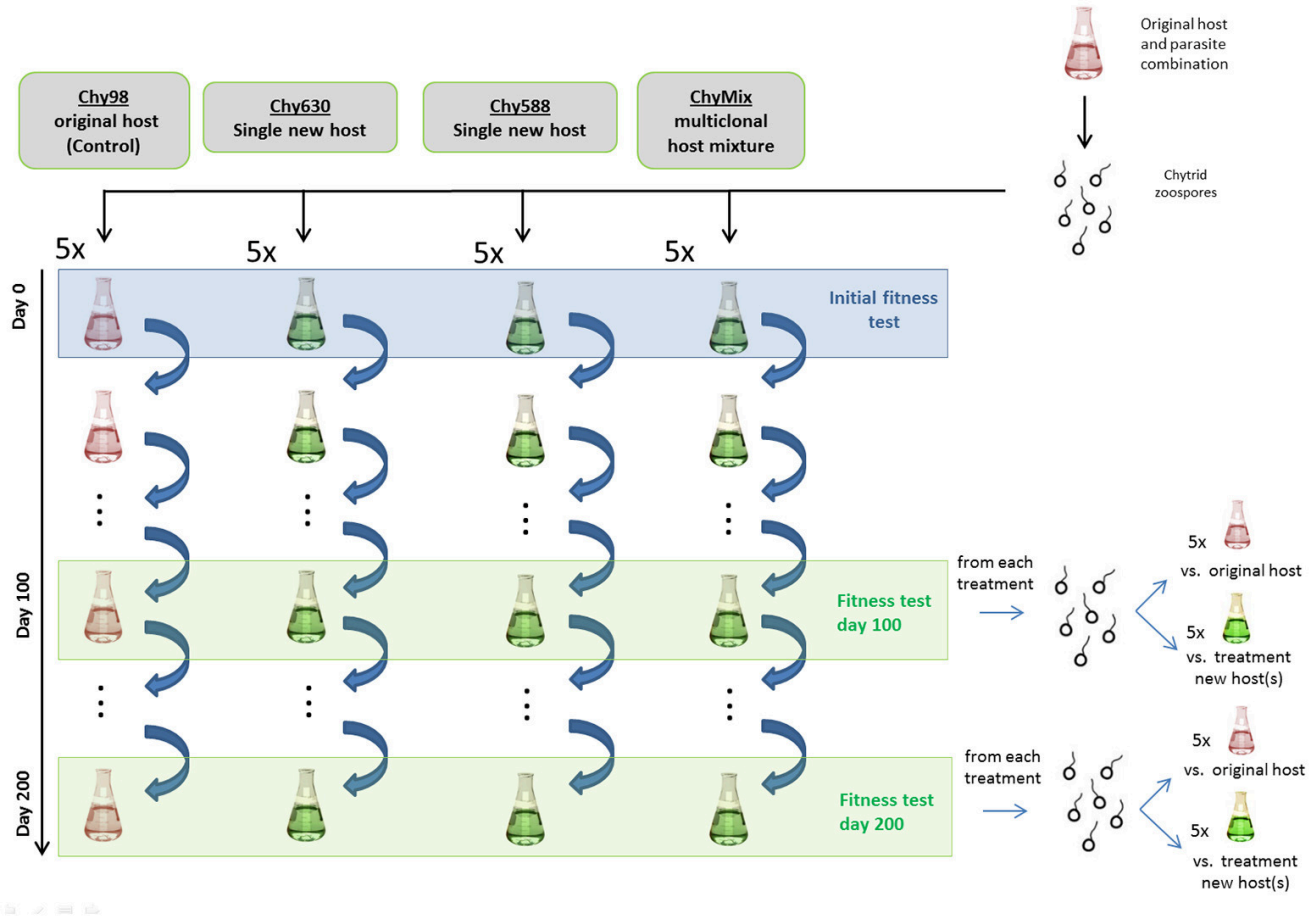
Schematic representation of the experimental setup.

A schematic representation of the experimental setup is provided in Figure 1. Ten days before the start of the experiment, a culture of the original host (i.e., NIVA-CYA98) was infected with the chytrid parasite and incubated at 20°C and 20 μmol photons m^−2^ s^−1^. After 10 days, a purified zoospore suspension was obtained by sequential filtration through sterile 10 and 5 μm nylon meshes and a 3 μm polycarbonate filter. The purified zoospore suspension was split among twenty 50 mL flasks, representing four different serial passage lines, each replicated five times. Serial passage lines (maintained under the above temperature and light conditions) provided different host compositions (Figure 1). For simplicity, we refer to each serial passage line as Chy-x, where x is the host strain number (Table S1) used to maintain the parasite in each line. The first line (Chy98) consisted of the original host and served as a control. The second (Chy630) and third lines (Chy588) were used to assess the ability of the parasite to adapt to suboptimal monoclonal hosts (NIVA-CYA630 and NIVA-CYA588, respectively; one replicate of Chy588 line was lost during passage). Lastly, we established an additional serial passage line (ChyMix) providing a multiclonal mixture of hosts in equal proportions to test whether host diversity can hamper parasite adaptation (six strains, see Table S1; original host was not included). For each host strain, filament densities were correlated with optical density at 750 nm before the start of the experiment and used to provide an initial filament density of 6,000 mL^−1^ in all lines. In the multiclonal line, each host strain was added in equal proportions to obtain the desired filament density. Individual parasite populations were maintained over 200 days by refreshing the cultures every 2 weeks. This was done by transferring 5 mL from the 2 week-old culture (>100,000 zoospores) into fresh host suspensions, thereby resetting host densities.

### Fitness tests

An initial fitness test was conducted to assess the performance of the parasite on each host composition at the beginning of the experiment. Two additional fitness tests were conducted after 100 and 200 days of experimental evolution, respectively. Here, individual parasite populations from each serial passage line were challenged against (1) their respective novel host(s) and (2) the original host (i.e., NIVA-CYA98). All fitness tests were performed under identical conditions: 2-week pre-acclimated host suspensions (50 mL; 6,000 filaments mL^−1^) were infected with zoospores stemming from each passage line. Zoospores were obtained from each passage line by sequential filtration, quantified under an inverted microscope using a Sedgewick Rafter chamber after fixation of a 1 ml aliquot with acid Lugol, and added into the fresh host cultures to provide a final concentration of 2,600 zoospores ml^−1^. The resulting infection cultures were incubated for 7 days at 20°C and 20 μmol photons m^−2^ s^−1^. Aliquots (2 mL) were sampled daily, fixed in 2% formaldehyde stored at 4°C, and analyzed within 2–4 weeks. All sample identities were blinded before analysis, except those from the initial fitness tests. Three different parasite traits were investigated: prevalence of infection, intensity of infection, and size of mature/empty sporangia after methods described in Agha et al. (2018). In brief, prevalence of infection was determined from daily collected samples as the proportion of infected filaments after screening 200 filaments per sample. Intensity of infection (the mean number of infections present on single infected hosts) was determined after examining 200 infected filaments per sample. Higher intensity of infection would indicate increased affinity of the parasite toward the cell surface of the host. Mean sporangial volumes were estimated from 40 empty or mature (i.e., fully developed) sporangia per sample by measuring their two semi-axes and assimilating their shape as rotational ellipsoids. In case multiple sporangia were present on the filament, only the biggest one was measured. Sporangial volume was used as a tentative proxy for reproductive output, as bigger sporangia imply more zoospores produced (Bruning, 1991b; Gerphagnon et al., 2013). Intensity of infection and size of sporangia were determined on samples from day 7 only, in order to ensure well-established levels of infection and fully developed/empty sporangia. Overall parasite fitness was expressed as transmission rate, based on the formulation by May and Anderson (1983):

(1) $$\begin{array}{l} Ro=\beta N/\alpha +\;b\;+\;\nu \end{array}$$

where *Ro* is the number of infections caused by a single primary infection, β is the parasite transmission rate, N is the density of hosts (kept constant in our experiment), α is the parasite virulence (equal to 1 here, as every infection is lethal), *b* is the rate of parasite independent mortality (assumed constant and negligible), and ν is the host recovery rate (equal to zero here). Therefore, under the standard conditions provided in the fitness tests, changes in parasite fitness could be attributed solely to changes in transmission rates. Transmission rates were estimated by logistic regression of the data reflecting the development of prevalence of infection over 7 days.

### Statistical analyses

Parasite performance was evaluated against different host compositions (vs. original/vs. new/vs. mix) and over evolutionary time. To evaluate potential adaptation of every parasite population to their respective new host(s) in each passage line, fixed effects of evolutionary time and host type (original/new host) were tested on each fitness parameter (transmission rate, intensity of infection and size of sporangia). This was done by conducting two-way ANOVAs, comparing parasite performance before and after 100 (or 200) days of experimental evolution. Data on sporangia needed to be log-transformed to meet distributional assumptions of the residuals. In addition, to test for parasite attenuation, differences in the performance of each evolved parasite population against its common original host were evaluated after 100 (or 200) days of experimental evolution. This was done by conducting one-way ANOVAs and subsequent Dunnett tests for multiple comparisons, testing for differences in the performance of each evolved parasite population against the non-evolved (control) one. All statistical analyses were performed using RStudio (v.0.99.903).

## Results

**Figure 2:**
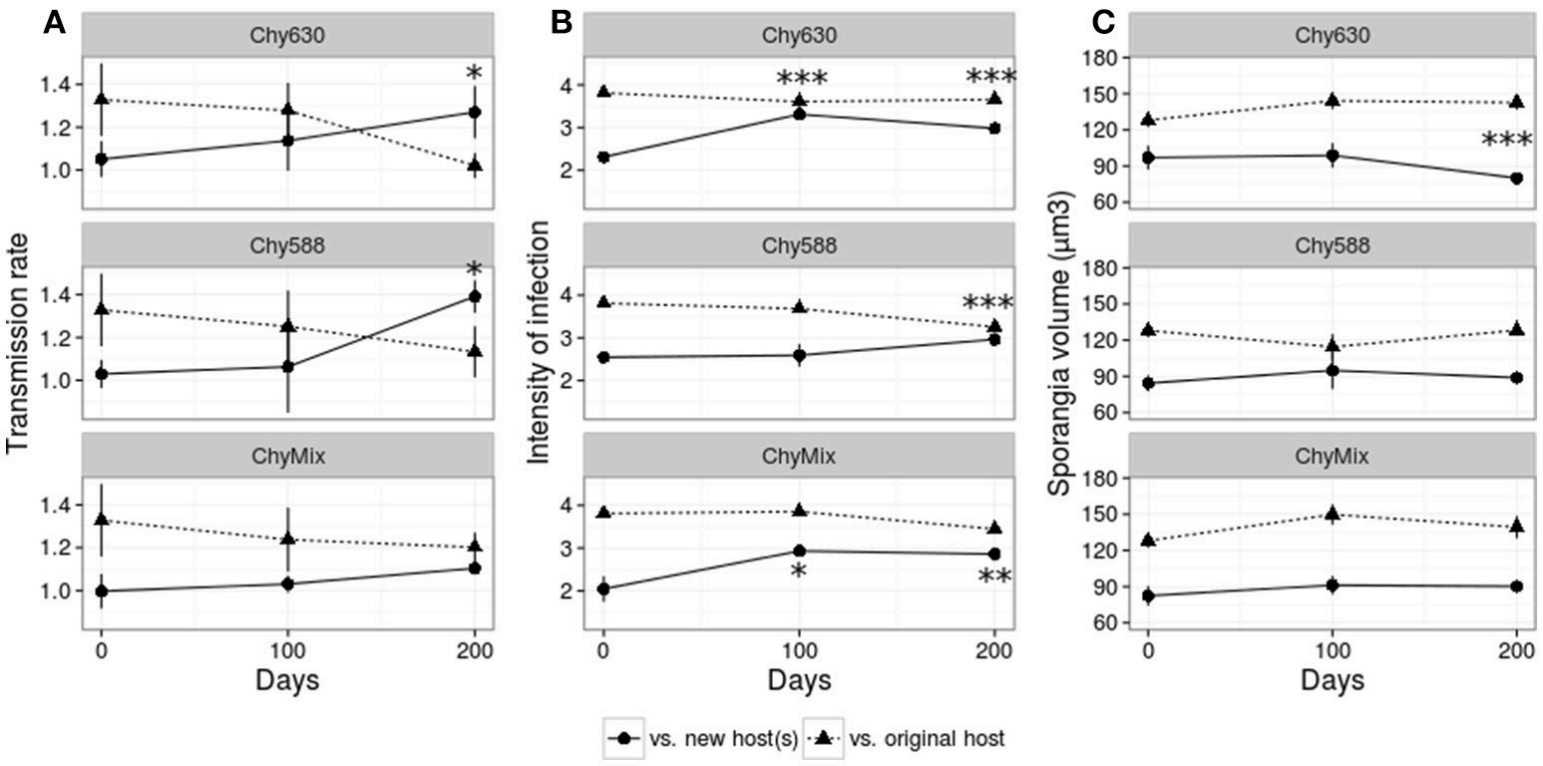
Performance of the parasite serial passage lines Chy630, Chy588 and ChyMix in terms of **(A)** transmission rate, **(B)** intensity of infection, and **(C)** size of mature/empty sporangia against their respective new host(s) (continuous line) and the original host (dashed line). Asterisks mark significant evolutionary time × host type interaction terms. Error bars represent std. error.

Figure 2 shows the performance of the different parasite populations infecting their respective new host(s) and infecting the original host. In the initial fitness test, the parasite consistently scored higher in all fitness parameters on the host it was maintained on, than on novel hosts (Figure 2, day 0). Adaptation of each evolving parasite population to the new host(s) was evaluated along the course of the experiment. Adaptation can be pictured as crossing parasite reaction norms when expressing parasite performance against new and original host(s) over evolutionary time [i.e., statistically significant interactions between evolutionary time and host type (original/new host)]. When expressing parasite fitness as transmission rate, parasite populations maintained on new monoclonal hosts showed adaptation to their respective new host (Chy630 or Chy588) after 200 days of evolution (Figure 2A; see significant interactions in Table 2). Such adaptation was not observed in the multiclonal treatment (Figure 2A, Table 2), where the parasite was maintained on a heterogeneous mixture of host genotypes each of which produced a different set of oligopeptides (Table 1). In contrast, increases in intensity of infection were observed after 100 and 200 days in multiclonal lines and in passage line Chy630 and, after 200 days in line Chy588 (Figure 2B, see significant interactions in Table 2). Regarding volume of mature/empty sporangia, no significant interactive effects were found, except for line Chy630, where parasites produced smaller sporangia when infecting the new host after 200 days of experimental evolution (Figure 2C, Table 2).

**Table 2:** Results of two-way ANOVAs testing for fixed effects of evolutionary time, host type and their interaction for each parasite evolution line and fitness parameter.

	**Chy630**		**Chy588**		**ChyMix**	
	**F0 vs. F100**	**F0 vs. F200**	**F0 vs. F100**	**F0 vs. F200**	**F0 vs. F100**	**F0 vs. F200**
**TRANSMISSION RATE**						
Time	0.895	0.710	0.896	0.500	0.819	0.928
Host	0.138	0.900	0.156	0.675	**0.041**	**0.049**
Time × Host	0.619	**0.037**	0.740	**0.036**	0.617	0.263
**INTENSITY OF INFECTION**						
Time	**<0.001**	**<0.001**	0.787	0.521	**0.012**	0.210
Host	**<0.001**	**<0.001**	**<0.001**	**<0.001**	**<0.001**	**<0.001**
Time × Host	**<0.001**	**<0.001**	0.569	**<0.001**	**0.021**	**0.004**
**SIZE OF SPORANGIA**						
Time	0.301	0.861	0.885	0.676	0.060	0.190
Host	**<0.001**	**<0.001**	**0.007**	**<0.001**	**<0.001**	**<0.001**
Time × Host	0.409	**<0.001**	0.258	0.697	0.404	0.809

*F0, F100, and F200 stand for fitness after 0, 100, and 200 days of experimental evolution, respectively. Significant p-values are depicted in bold*.

**Figure 3:**
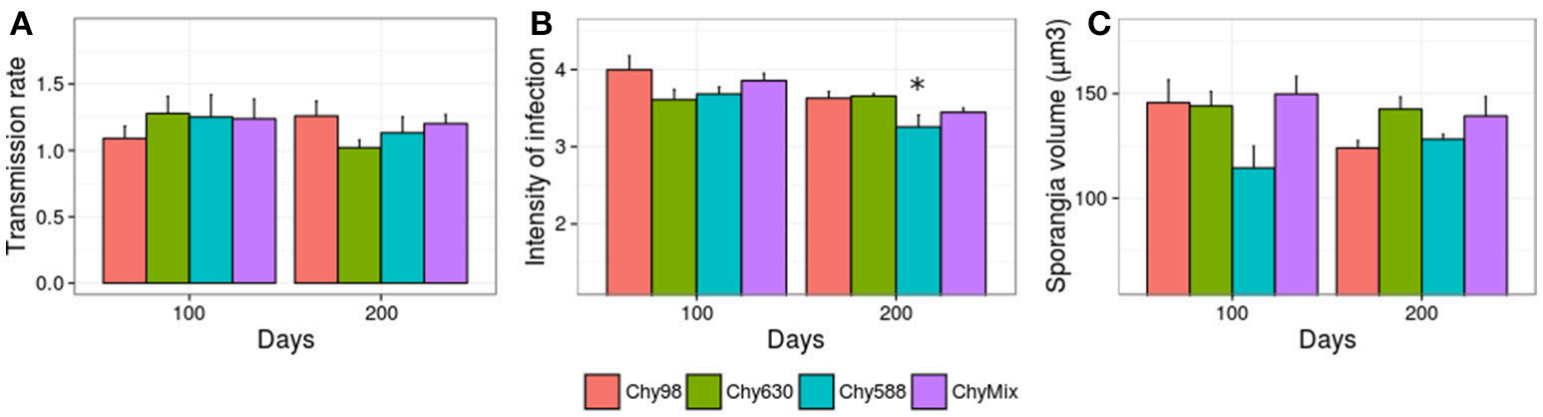
Performance of each evolving parasite population in terms of **(A)** transmission rate, **(B)** intensity of infection, and **(C)** size of mature/empty sporangia against the original host after 100 and 200 days of experimental evolution. Asterisks depict significant differences between the parasite serial passage line and the control line (Chy98) (Dunnett test). Error bars represent std. error.

In addition to adaptation, parasite attenuation was evaluated, i.e., the potential loss of performance against the original host upon adaptation to novel ones. Specifically, parasite performance of each evolving parasite population (Chy630, Chy588, and ChyMix) was scored on the original host, and compared with the performance of the non-evolved parasite population (Figure 3). There was no evidence for parasite attenuation with the exception of intensity of infection in Chy588, with significant reductions after 200 days of experimental evolution, compared to the control line maintained on the original host (Figure 3B).

## Discussion

The results of the serial passage experiment demonstrate the ability of the chytrid parasite to adapt rapidly to single new hosts. Although the parasite initially showed reduced fitness against novel monoclonal hosts, evolutionary change occurred quickly and fitness increase was evident after 200 days. This illustrates the strength of selection imposed by new host environments and exemplifies the ability of parasites to adapt rapidly to them. Increased chytrid performance can result from genetic change, phenotypic plasticity, or some combination of the two. Considering genetic change, the fact that our experiment was initiated using a single parasite strain would point toward mutation as the mechanism leading to genetic variation, upon which selection operates. In addition to selection, genetic drift could have potentially led to genetic changes as a result of parasite population bottlenecks caused by serial transfers. However, bottlenecks were systematically avoided by transferring over 100,000 zoospores every passage, a population size that is orders of magnitude above reported thresholds, below which genetic drift starts to shape allele frequencies (Poulin and Morand, 2000). Moreover, drift produces non-adaptive random patterns, characterized by divergent results across replicates, which contrasts with the consistent evolutionary response obtained here across replicate passage lines. An alternative possibility is that adaptation to new hosts was due to phenotypic plasticity, i.e., the ability of a genotype to produce different phenotypes as the environment changes; for example through mechanisms such as differential gene expression. There is increasing evidence that parasites rely on a variety of flexible (plastic) traits to adapt to new environments (Mideo and Reece, 2012). For instance, parasite traits involved in replication (such as life cycle duration or burst sizes; Gautret et al., 1995; Leggett et al., 2013) or resource allocation to sexual/asexual modes of reproduction (e.g., Pollitt et al., 2011) have been shown to exhibit plastic responses that maximize fitness across changing environments. Interestingly, whereas host-parasite compatibility is often assumed to have a genetic basis, it has also been showed to be shaped by phenotypic plasticity (Little et al., 2006). Identifying the nature and quantifying the contribution of these mechanisms to chytrid adaptation to new hosts remains a key open question to better understand the epidemiology of chytridiomycosis.

In spite of rapid adaptation of the parasite to novel monoclonal hosts, fitness improvement was not observed in the multiclonal treatment. This demonstrates that cyanobacterial genetic diversity prevents, or at least slows, parasite adaptation. Similar effects of genetic diversity have been observed for a diatom population infected by a chytrid fungus (De Bruin et al., 2008). In water flea *Daphnia*, more genetically diverse populations showed higher resistance against a microsporidian parasite (Altermatt and Ebert, 2008). These findings support the general idea that high host genetic diversity reduces disease prevalence (King and Lively, 2012). In genetically diverse host populations, after a successful primary infection, parasites of the next generation encounter different host genotypes, whose optimal infection may require different adaptations. In our experiment, the multiclonal treatment did not include any fully resistant strain that could act as a transmission dead-end. Instead, we attribute unchanged transmission to an overall inability of chytrids to efficiently overcome defenses of hosts with dissimilar genetic backgrounds (Sønstebø and Rohrlack, 2011).

Our experiment tested for asymmetric evolution (i.e., host remained in evolutionary stasis). However, in nature, hosts and parasite selection is reciprocal. If cyanobacterial genetic diversity protects against disease spread, it is tempting to hypothesize that chytrid parasitism can induce diversification in cyanobacteria. Diversification of marine cyanobacteria as a co-evolutionary response against bacteriophages has been demonstrated experimentally (Marston et al., 2012), and indications of chytrid parasitism promoting increased genetic diversity in natural diatom populations further support this idea (Gsell et al., 2013). In nature, long-term reciprocal adaptation between chytrids and their cyanobacterial hosts has been proposed to lead to a Red Queen co-evolutionary dynamics (Sønstebø and Rohrlack, 2011; Kyle et al., 2015), in which parasites impose strong negative frequency-dependent selection on host populations (i.e., common host genotypes are more strongly decimated by matching parasites). Such dynamics buffers competitive differences between conspecific genotypes and leads thereby to long-term maintenance of genetic diversity in host and parasite populations (Hamilton et al., 1990).

Natural cyanobacterial populations display a mosaic structure of highly dynamic subpopulations with regard to various polymorphisms, such as the production of hypervariable secondary metabolites (e.g., oligopeptides; Welker and von Dohren, 2006; Agha and Quesada, 2014). Maintenance of such polymorphisms over decades (Rohrlack et al., 2008), despite associated high metabolic costs for the host (Amoutzias et al., 2008) is consistent with Red Queen evolutionary models and speaks for the strength of the selective forces fueling such diversity. Indeed, parasites impose strong selection on their hosts and there are indications that cyanobacterial oligopeptides are involved in resistance against chytrid parasites (Rohrlack et al., 2013), by analogy with other hypervariable cyanobacterial traits conferring resistance to phages (Avrani et al., 2011). Hypervariable oligopeptide production is largely attributable to relaxed specificity in the incorporation of amino acids during non-ribosomal biosynthesis, leading to the existence of multiple structurally similar oligopeptide variants with diverse bioactive properties (Nagarajan et al., 2013). Interestingly, genetic regions responsible for such amino acid promiscuity are often under positive selection (e.g., Tooming-Klunderud et al., 2008; Rounge et al., 2010), suggesting that oligopeptide chemical diversity might be evolutionarily promoted. This, together with other mechanisms, including frequent horizontal gene transfer, module reshuffling or recombination in oligopeptide-encoding gene-clusters strongly suggests the existence of different mechanisms in cyanobacteria that generate metabolic diversity with minimal genetic changes and is consistent with host diversification predicted by Red Queen co-evolutionary models (Agha and Quesada, 2014). Further experiments will aim at directly relating chemical diversification in cyanobacteria and the co-evolutionary interaction with their (chytrid) parasites.

In our experiment, no evidence for attenuation was found. Adaptation often results in a specialization process, where parasite performance against new hosts trades-off with that against former ones. Although this may be the case sometimes, especially when different hosts represent different species (Ebert, 1998), adaptive changes may also result in unchanged or even increased performance on foreign hosts. Little et al. (2006) argued that a generalization about the occurrence of attenuation between host genotypes (i.e., belonging to the same species) is difficult to make. A mechanistic understanding of the infection process seems necessary to make better predictions about the evolution of host ranges and specialism in this host-parasite system.

As chytrid infection always leads to the death of the host (virulence equals 1), increases in chytrid fitness are solely the result of changes in transmission. However, parasite transmission is a composite variable (*sensu* Antolin, 2008) that is the result of different traits, such as the affinity of the parasite to encyst on its host, the number of zoospores produced per sporangium, its sporulation/generation time, or the infective lifetime of zoospores (Bruning, 1991a,b). In our experiment, we first addressed the encystment phase of the infection. Consistent increases in intensity of infection observed across passage lines (i.e., higher parasite encystment on hosts) indicate that traits involved in cell-to-cell recognition are evolutionarily highly dynamic in this host-parasite system. Knowledge from other fungi (e.g., Levitz, 2010; Petre and Kamoun, 2014) suggests that these traits likely involve lectins and complex carbohydrates at the chytrid and cyanobacterial cell surfaces, respectively. In analogy, cell surface traits of marine cyanobacteria have been shown to be encoded in hypervariable genomic islands, which are strongly related to viral susceptibility (Avrani et al., 2011). We predict the existence of analogous genomic co-evolutionary hotspots in *Planktothrix*, which could be used as diagnostic tools for mapping host-parasite specificity. Secondly, in light of the correlations between sporangial size and the number of produced zoospores (Bruning, 1991b; Gerphagnon et al., 2013; Van den Wyngaert et al., 2014), we addressed sporangial size as a tentative proxy for the efficiency with which the parasite exploits it host after encystment. However, we did not observe any increase, but, in fact an occasional decrease in sporangial sizes over time. Higher intensity of infection arguably leads to increased competition between co-infecting zoospores for host resources, which impacts final sporangial sizes, as suggested by negative correlations between intensity of infection and final sporangial sizes reported elsewhere for this system (Agha et al., 2018). In that same study, both sporangia and zoospores sizes varied along temperature gradients, leading to the hypothesis that chytrids may exploit trade-offs between size of sporangia and zoospores to stabilize their reproductive output and maximize transmission across changing environments (Agha et al., 2018). Highly dense and virtually unlimited host conditions provided in the present experiment might have selected for short parasite generation times (i.e., small sporangia that display short maturation times) and maximal reproductive output (i.e., relatively smaller zoospores) to maximize transmission. This is consistent with demographic and epidemiological models predicting selection for short generation times and early transmission in expanding parasite populations (Bull and Ebert, 2008). However, zoospores rely on internal energy reserves to find new hosts and reductions in zoospore size thus come at the expense of shorter infective lifetimes. Under natural conditions, host densities are typically lower and far more variable than in our experiment and, therefore, the inability to find a suitable host arguably constitutes a major bottleneck for chytrid transmission. We argue that investing in long infective lifetimes is likely the dominant strategy in the wild. Testing these predictions demands addressing additional parasite traits that act as component variables of transmission, such as sporulation times, and zoospore size and longevity. On top of this, further studies should address how natural conditions, including environmental variation and additional biotic interactions (e.g., with heterotrophic bacteria) affect chytrid-host evolution (Wolinska and King, 2009).

All in all, our results demonstrate the adaptive potential of chytrid parasites and support the finding that genetic diversity protects phytoplankton populations from disease (De Bruin et al., 2008). High susceptibility to disease among low diversity host populations can be attributed to the ability of parasites to rapidly adapt to them, quickly leading to high transmission among homogeneous hosts. Conversely, we show that host genetic diversity, in addition to hindering efficient parasite transmission between heterogeneous hosts, grants resistance to disease by hampering parasite adaptation.

## Author contributions

RA: conceived the study; RA, AG, TR, and JW: designed the experiment; RA and AG: conducted the experiment and analyzed the data; RA: wrote the manuscript with input from JW. All authors read and commented on the final version of the manuscript.

### Conflict of interest statement

The authors declare that the research was conducted in the absence of any commercial or financial relationships that could be construed as a potential conflict of interest.
