# Supplementary material for: Adaptation of a Chytrid Parasite to Its Cyanobacterial Host Is Hampered by Host Intraspecific Diversity
Source: Front Microbiol. 2018 May 8;9:921. doi: 10.3389/fmicb.2018.00921 (PMC5952108; doi:10.3389/fmicb.2018.00921)
Supplement: Supplementary file 1 [file Table_1.docx]

| Strain name | Taxonomic affiliation | Site of isolation | Date of isolation | Treatment |
| --- | --- | --- | --- | --- |
| Chy-Kol2008 | *Rhizophydium megarrhizum* | Lake Kobotnvatet (Norway) | 2008 |  |
| NIVA-CYA98 | *Planktothrix rubescens* | Lake Steinsfjorden (Norway) | 1982 | control |
| NIVA-CYA630 | *Planktothrix agardhii* | Lake Lyseren (Norway) | 2008 | mono, multi |
| NIVA-CYA588 | *Planktothrix agardhii* | Lake Langer See (Germany) | 2005 | mono, multi |
| NIVA-CYA557 | *Planktothrix agardhii* | Lake Langer See (Germany) | 2005 | multi |
| NIVA-CYA578 | *Planktothrix agardhii* | Lake Langer See (Germany) | 2005 | multi |
| NIVA-CYA580 | *Planktothrix agardhii* | Lake Langer See (Germany) | 2005 | multi |
| NIVA-CYA562 | *Planktothrix agardhii* | Lake Langer See (Germany) | 2005 | multi |

Table S1. List of chytrid parasite (*Rhizophydium megarrhizum*) and cyanobacteria host (*Planktothrix* spp.) strains used. The column “treatment” indicates in which evolutionary treatment(s) each cyanobacterial strain was used; mono: monoclonal treatment; multi: multiclonal treatment.
